# Compound lidocaine/prilocaine cream combined with tetracaine prevents cough caused by extubation after general anaesthesia: a randomised controlled trial

**DOI:** 10.1186/s12871-022-01964-3

**Published:** 2023-01-03

**Authors:** Erfei Zhang, Xiaoying Zhao, Ting Li, Min Wang, Jie gao, Hailiang Zhang, Ying Li, Lei Zhang, Taiyang Li

**Affiliations:** 1 Department of Anesthesiology, The Affiliated Hospital of Yan’an University, Yan’an 716000, Xi’an, Shaanxi Province People’s Republic of China; 2 Department of Anesthesiology, Shanxi Province, Second Hospital of Shanxi Medical University, Taiyuan, 030001 People’s Republic of China; 3 Department of Anesthesiology, Shaanxi Province, Northwest Women’s and Children’s Hospital, Xi’an 710061, People’s Republic of China

**Keywords:** Tetracaine, Compound lidocaine/prilocaine cream, Cough, Extubation, General anaesthesia

## Abstract

**Background:**

Coughing caused by tracheal extubation is common following general anaesthesia. Heavy aerosol production by coughing during recovery from general anaesthesia in patients with respiratory infections (especially COVID-19) may be one of the highest risk factors for infection in healthcare workers. The application of local anaesthetics to the endotracheal tube is an effective method to reduce coughing. The most commonly used anaesthetics are compound lidocaine/prilocaine cream and tetracaine spray. However, coughing still occurs when the two anaesthetics are used alone. We speculated that the application of compound lidocaine/prilocaine combined with tetracaine spray would better prevent coughing caused by tracheal extubation.

**Methods:**

Patients scheduled for laparoscopic cholecystectomy or cholecystectomy combined with common bile duct exploration under general anaesthesia were randomly assigned to Group C (saline spray), Group L (2 g compound lidocaine/prilocaine cream contains 5 mg of lidocaine and 5 mg prilocaine)), Group T (tetracaine) and Group F (compound lidocaine/prilocaine cream combined with tetracaine). The incidence of coughing, the endotracheal tube tolerance assessment, the incidence of agitation, the active extubation rate, the incidence of postoperative pharyngeal pain and the incidence of postoperative cough were recorded and analysed. Systolic blood pressure (SBP), diastolic blood pressure (DBP), heart rate (HR), and the plasma concentrations of epinephrine and norepinephrine were measured immediately before extubation and 1 min after extubation.

**Results:**

A total of 211 patients were randomly assigned to Group C (53 cases), Group L (52 cases), Group T (52 cases) and Group F (54 cases). The primary result is assessment of the incidence of cough. The patients emerged from general anaesthesia, 96% of Group C had cough, which was significantly reduced in Group L (61.5%, *P* < 0.001), Group T (75%, *P* < 0.05) and Group F (22.2%, *P* < 0.001). Group F had a significantly reduced incidence of cough compared to Group L and Group T (*P* < *0.05* or *P* < 0.01, respectively). The secondary results were assessed. The endotracheal tube tolerance score in Group C ((1, 3) 4, *P* < 0.001) was higher than Group L ((0, 1) 2), Group T ((0, 1.25) 3) and Group F ((0, 0) 1). Group F had a significantly lower score than Group L and Group T (*P* < 0.05, *P* < 0.01, respectively). The incidence of agitation and the active extubation rate were also higher in Group C (96.2% and 71.7%, respectively, *P* < 0.001) than Group L (48.1% and 15.4%, respectively), Group T (61.5% and 26.9%, respectively) and Group F (17.3% and 7.7%, respectively). Blood pressure, HR and plasma concentrations of epinephrine and norepinephrine were significantly higher in Group C than in all other groups at the time of extubation and 1 min after extubation (*P* < 0.001). Group F exhibited significantly reduced blood pressure, heart rate and plasma concentrations of epinephrine and norepinephrine compared to Group L and Group T (*P* < 0.05, *P* < 0.01 or *P* < 0.001, respectively). The incidence of postoperative pharyngeal pain and the incidence of postoperative cough were not significantly different among the groups.

**Conclusions:**

Compound lidocaine/prilocaine cream combined with tetracaine may be a more effective approach for preventing coughing and stabilising circulation during extubation following general anaesthesia. This may play an important role in preventing medical staff from contracting respiratory infectious diseases.

**Trial registration:**

Chinese Clinical Trial Registry: ChiCTR2200058429 (registration date: 09–04-2022) “retrospectively registered”.

## Introduction

Coughing caused by endotracheal tubes in general anaesthesia is one of the most common reflexes in clinical medicine, especially when the trachea is extubated and the incidence is between 38 and 96% [1]. Patients with respiratory infections generate significant aerosols when coughing [2]. One study suggested that extubations generated mean aerosol concentrations that were statistically comparable to coughing. A patient cough during extubation, produced a detectable aerosol that was 15-fold greater than intubation [3]. Transmission of severe acute respiratory syndrome coronavirus 2 (SARS-CoV-2) occurs primarily via contact with droplets, fomites and, to a lesser degree, aerosol generation [4]. Therefore, reducing the incidence of cough during recovery from general anaesthesia is essential to reduce the risk of respiratory virus infection of medical staff. Irritation to the epiglottis and airway mucosa by the endotracheal tube is the anatomical cause of coughing and sore throat. Compound lidocaine/prilocaine cream application to tracheal tubes and tetracaine spray on airway mucosa are the most commonly used methods to prevent cough during extubation following general anaesthesia [5, 6]. However, our previous clinical work showed that nearly 50% of patients still cough. Tetracaine has strong mucosal permeability and a good effect on mucosal surface anaesthesia [7], but tetracaine spraying of the airway surface is not a very convenient clinical application, especially for patients with respiratory infectious diseases. One type of thermosensitive hybrid hydrogel containing tetracaine changed the viscoelastic properties at the nasal mucosa temperature (32 °C) and thus decreased the number of free anesthetic molecules available to cross the epithelial barrier [8]. Therefore, the anesthetic effect time was prolonged by thermosensitive hybrid hydrogel tetracaine compared with tetracaine directly loaded in the hydrogel. However, it is difficult to obtain the thermosensitive hybrid hydrogel tetracaine in the clinical medicine. Improving the convenience of tetracaine use is a clinical problem that needs to be solved. We referred to our anecdotal clinical observations that enhanced the effect of tetracaine on airway surface mucosa is the application of water-soluble compound lidocaine/prilocaine cream to the front end of the tracheal tube followed by the spraying of tetracaine on the compound lidocaine. The possible mechanism is that the water-soluble compound lidocaine/prilocaine cream increases the amount of tetracaine contact with the airway mucosal surface compared to the simple tetracaine spray. Therefore, we speculated that compound lidocaine/prilocaine cream combined with tetracaine coating of the surface of the endotracheal tube would reduce the coughing response during recovery from general anaesthesia. Our study performed a randomised controlled trial (RCT) on this application.

## Methods

### Trial design

The Affiliated Hospital of Yan’an University, China organised this RCT. The trial was performed according to the CONSORT-2010 guidelines. The Ethics Committee of The Affiliated Hospital of Yan’an University approved the study protocol (NO. 2020042), and all subjects provided written informed consent before participating in the trial.

### Participants and setting

**Fig. 1:**
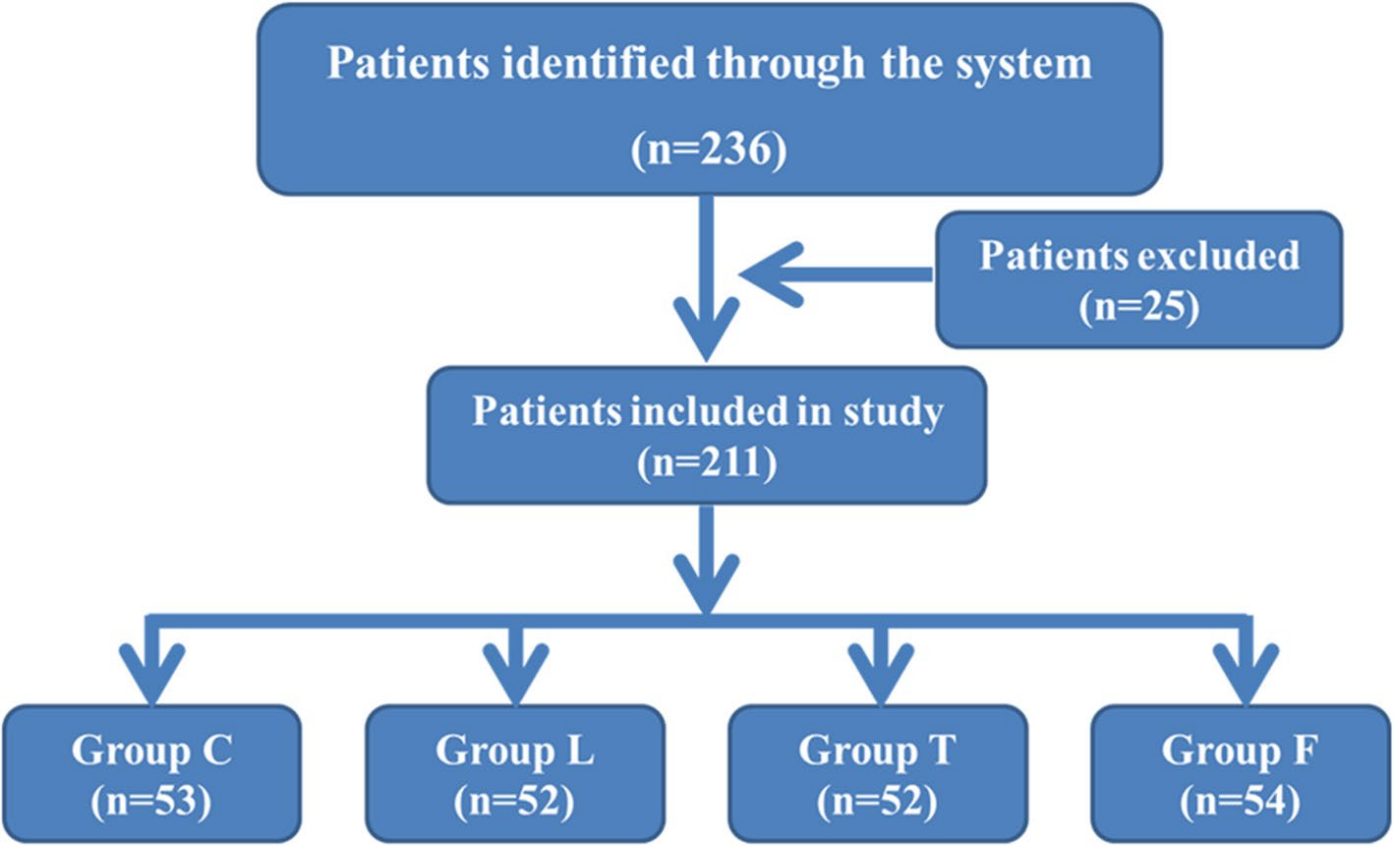
Consolidated Standards of Reporting Trials flow diagram A total of 236 patients were included in the study. 25 patients were excluded according to the exclusion criteria. A total of 211 patients were randomly divided into 4 groups, 53 patients in group C, 52 patients in group L, 52 patients in group T and 54 patients in group F

Patients were included in the trial when they were 18 ~ 64 years old and scheduled for laparoscopic cholecystectomy or cholecystectomy combined with common bile duct exploration under general anaesthesia using endotracheal tube intubation. The following major exclusion criteria were used: American Society of Anaesthesiologists (ASA) grade greater than III; preoperative chronic pharyngitis, cough or other upper respiratory tract lesions; concurrent hypertension with or without drug therapy; difficult airway; allergies to lidocaine, tetracaine, or any other ingredients in the test product; intraoperative bronchospasm; operation time greater than 2.5 h; intraoperative bleeding (> 300 ml). We randomly assigned the patients to placebo (normal saline), compound lidocaine/prilocaine cream, tetracaine spray, or compound lidocaine/prilocaine cream combined with tetracaine treatment at a 1:1:1:1 ratio. The primary end points included the overall incidence of cough, the endotracheal tube tolerance cough and cardiovascular reactions during extubation after anaesthesia during the recovery period, the sore throat within the first 24 h after extubation. Figure 1 shows a flowchart for the assignment of participants in the study.

### Randomisation and blinding

A total of 236 random numbers were generated by IBM SPSS Statistics 25 (IBM Corp. Released 2017. IBM SPSS Statistics for Windows, Version 25.0. IBM Corp, Armonk, NY, USA), and the software randomly divided the 236 numbers into four groups. Cases were enrolled according to the order of enrolment time corresponding to random numbers from small to large, and a random number corresponded to the admission ID number of the patient. A full-time anaesthesiologist (Investigator A) performed these assignments. The patient entered the operating room, and investigator A induced general anaesthesia according to the conventional method. Tracheal intubation was performed after smearing and/or spraying the tracheal tube according to the enrolment information. After completion of tracheal intubation, another anaesthesiologist (Investigator B) performed anaesthesia management according to conventional methods and collected data until the end of the study. All of the collected data were handed over to Investigator A for sorting into different groups, and Investigator C performed statistical analyses. The patients and Investigators B and C were all blinded to the grouping information.

### Intervention

Anaesthesiologist A treated the surface of the tip of the endotracheal tube according to the random number table. In Group C, normal saline (2 ml) was evenly sprayed onto the front end of the tracheal tube up to the two black marked lines near the cuff. In Group L two grams of compound lidocaine/prilocaine cream (compound lidocaine/prilocaine cream, 10 g, containing 25 mg each of lidocaine and prilocaine, Tongfang Pharmaceutical Group Co., Ltd. Beijing China) was evenly applied onto the front end of the tracheal tube up to the two black marked lines near the cuff. In Group T, 2 ml of 10% tetracaine (tetracaine hydrochloride for injection, 50 mg, Chengdu Zhengkang Pharmaceutical Co., Ltd. Chengdu, China) was evenly sprayed onto the front end of the tracheal tube up to the two black marked lines near the cuff. In Group F, two grams of compound lidocaine/prilocaine cream were first applied onto the front end of the tracheal tube up to the two black marked lines near the cuff then a small container with a spray function was used to evenly spray 2 ml of 10% tetracaine injection onto the front end of the tracheal tube where compound lidocaine adhered. The method of applying compound lidocaine/prilocaine cream onto the front end of the tracheal tube up to the two black marked lines near the cuff was performed according to a previous study [5]. The endotracheal tube sizes were selected according to our anaesthesiology department protocol (males, ID: 7.5–8.0 mm size; females, ID: 6.5–7.0 mm size), and according to the size of the glottis observed under the video laryngoscope. After ventilation, the anaesthesiologist (A) placed and fixed the endotracheal tube. Sufentanil, propofol and rocuronium bromide were used as the induction agents according to the sex and weight of the patients. Rocuronium bromide was the muscle relaxant, and remifentanil and propofol were used according to the weight and situation of the patients during the surgery. The depth of anaesthesia was measured by the bispectral index, (BIS, BIS™, Medtronic, Minneapolis, MN, USA) and the BIS was maintained at the 40 ~ 60 level. The depth of anaesthesia was maintained by intravenous anaesthesia using continuous infusions of remifentanil and propofol and intermittent intravenous injections of cisatracurium. All infusions were discontinued when the incision was closed. The postoperative analgesia in this study used a multimodal analgesic strategy, in which 40 mg sodium parecoxib was intravenously injected before skin incision, 20 ml of 0.2% ropivacaine was given at an intraperitoneal location, and ~ 1 ml/cm of 0.2% ropivacaine was given at the site of incision at the end of surgery. No patients required additional analgesics in the PACU.

### Parameter measurement

The primary outcome measure was the incidence of induced coughing due to endotracheal extubation. The definition of induced cough was patients with coughing induced by extubation during recovery from anaesthesia. Secondary outcome measures were the incidence of agitation and active extubation, endotracheal tube tolerance ranking and cardiovascular reactions prior to extubation and postoperative cough and postoperative pharyngeal pain. The degree of endotracheal tube tolerance was scored as follows: 0 = no response during breathing, including spontaneous and mechanical ventilation conditions; 1 = no response during breathing, including spontaneous and mechanical ventilation conditions, but slight action response to aspiration of sputum (inconspicuous coughing reaction); 2 = tolerance to mechanical ventilation, but moderate action response to aspiration of sputum (single coughing); 3 = tolerance to ventilation, severe coughing reaction (multiple coughs that lasted shorter than 5 s) caused by sputum aspiration; 4 = could not tolerate mechanical ventilation, severe coughing reaction caused by sputum aspiration; and 5 = extubation behaviour. Coughing was scored as follows: 0 = no cough; 1 = mild cough; 2 = moderate cough, multiple coughs that lasted shorter than 5 s; and 3 = severe cough, multiple coughs that lasted longer than 5 s. The definition of agitation was patient showed thrashing or violent behaviour with removal of tubes and tracheal tube during recovery from anaesthesia according to previous report [9]. The definition of active extubation was the patient tried to pull the tracheal tube out by hand, but it was not pulled out (special staff ensured that the tube was not removed) during recovery from anaesthesia. The definition of postoperative cough was more than 5 spontaneous coughs that lasted longer than 5 s within the first 24 h after extubation, as previously described [10]. Cardiovascular reactions prior to extubation included systolic blood pressure (SBP) (mmHg), diastolic blood pressure (DBP) (mmHg), heart rate (HR) (beats/min), plasma concentrations of epinephrine (E) (pmol/l) and norepinephrine (NE) (pmol/l). SBP, DBP, and HR were measured with a monitor. Blood samples were collected immediately before extubation and 1 min after extubation from the arm without an intravenous infusion, according to a previous report [10]. Concentrations of epinephrine and norepinephrine, were measured using a commercially available ELISA kit (IBL, Germany) and an ELISA plate reader (BioTek, Winooski, VT, USA).

### Conditions of endotracheal tube extubation

The following conditions were used for tracheal tube extubation: 1) spontaneous breathing tidal volume greater than 6 ml/kg; 2) breathing of air for at least 5 min with saturation of pulse oximetry (SPO_2_) not lower than 95%; 3) response to a shoulder tap and calling of the patient’s name, including opening of the eyes and nodding of the head.

### Sample size calculation

According to our previous pilot studies, the incidence of cough induced by extubation was 95% in Group C, 45% in Group L, 60% in Group T, and 15% in Group F. We set α = 0.05 and β = 0.25, with a sample drop-out rate of 15%. Using PASS 15, we calculated a minimum sample size of 59 cases in each group (a total of 236 cases).

### Statistical analysis

After testing for normality (Shapiro–Wilk test), the data were expressed as means ± SD and non-normally distributed data as median [interquartile range] or mean [95% CI], as appropriate. Measurements were calculated as means (± standard deviation). ANOVA was used to compare different groups. Non-parametric one-way ANOVA (Kruskal–Wallis test) was used for rating data. Pearson's chi-squared test was used to compare the categorised data in the groups. We set *p* < 0.05 as statistically significant. SPSS 25 was used to process the data.

## Results

### Patients

A total of 236 patients at the Affiliated Hospital of Yan'an University were enrolled in the randomised trial. Twenty-five patients were excluded from this study, including 6 cases in Group C (difficult airway: 2 cases; patients with concurrent hypertension: 4 cases), 7 cases in Group L (difficult airway: 2 cases; preoperative chronic pharyngitis: 2 cases; allergies to compound lidocaine/prilocaine cream: 1 case; patients with concurrent hypertension: 2 cases), 7 cases in Group T (difficult airway: 3 cases; allergies to compound lidocaine/prilocaine cream: 1 case; patients with concurrent hypertension: 2 cases; operation time longer than 2.5 h: 1 case), and 5 cases in Group F (difficult airway: 1 case; operation time longer than 2.5 h: 1 case; intraoperative bleeding > 300 ml: 1 case; patients with concurrent hypertension: 2 cases). The study started on March 1, 2020 and ended on December 31, 2020. There were no significant differences in the baseline characteristics (such as age, weight, BMI, sex, smoking, operation time, anaesthesia time and BIS) of the patients among the groups (Table 1).

**Table 1:** Patient characteristics

	**Group C**	**Group L**	**Group T**	**Group F**	***F/****χ*^***2***^	***p***
Age(year)	41.11 ± 9.811	43.71 ± 9.831	41.00 ± 9.770	42.81 ± 7.690	1.063	0.366
Weight(kg)	65.58 ± 10.11	65.60 ± 9.091	67.54 ± 9.881	67.02 ± 8.313	0.595	0.619
BMI	23.96 ± 4.617	23.83 ± 3.966	24.58 ± 4.747	24.43 ± 3.754	0.372	0.773
Sex (male n (%))	9(16.7%)	14(26.9%)	10(19.2%)	11(20.4%)	1.733	0.630
Smoking (n (%))	12 (22.6%)	12 (23.1%)	8 (15.4%)	13 (24.1%)	1.478	0.658
Operation time (minutes)	52.17 ± 23.79	46.23 ± 20.83	45.77 ± 25.37	49.37 ± ± 25.62	0.815	0.487
Anesthesia time (minutes)	67.17 ± 23.79	61.23 ± 20.83	60.77 ± 25.37	64.37 ± 25.62	0.815	0.487
BIS	48.04 ± 3.287	47.90 ± 4.146	46.40 ± 3.403	46.96 ± 3.458	2.476	0.063

## Primary outcomes

**Fig. 2:**
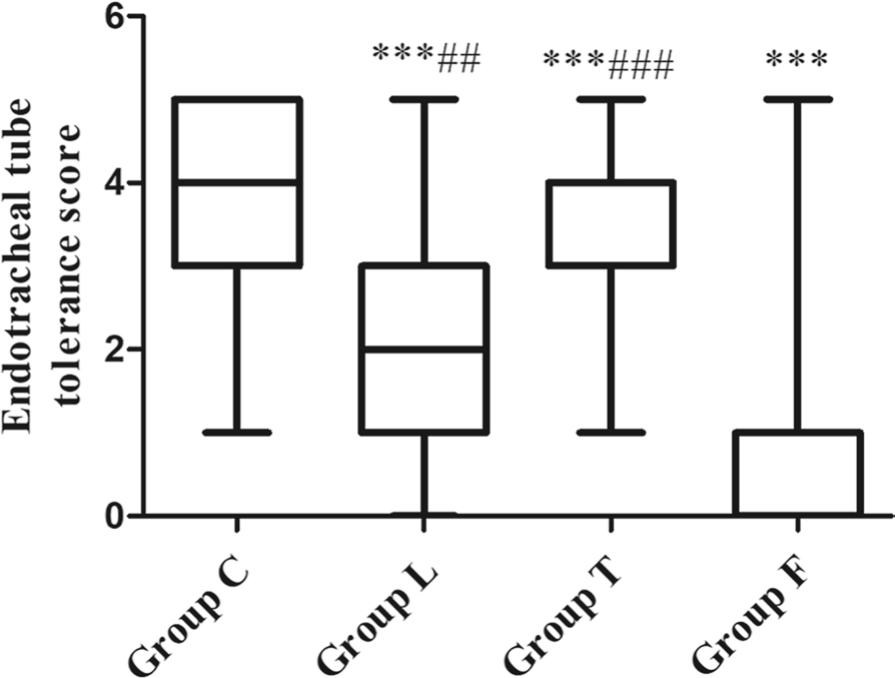
Comparison of tracheal tube tolerance between groups Endotracheal tubes were treated with saline, compound lidocaine/prilocaine cream, tetracaine, or compound lidocaine/prilocaine cream combined tetracaine as per randomization. The degree of endotracheal tube tolerance was scored as following: 0 = no response during breath including spontaneous and mechanical ventilation condition; 1 = no response during breath including spontaneous and mechanical ventilation condition, but slight action response to aspiration of sputum (inconspicuous coughing reaction); 2 = tolerance to mechanical ventilation, but moderate action response to aspiration of sputum(single coughing); 3 = tolerance to ventilation, severe coughing reaction (multiple coughing but lasted shorter than 5 s) caused by sputum aspiration; 4 = cannot tolerate mechanical ventilation, severe coughing reaction caused by sputum aspiration; 5 = extubation behavior. Data are expressed as median + up-interquartile range/down up-interquartile range. ^***^ *P* < 0.001 vs. group C; ^# #^ *P* < 0.01, ^###^*P* < 0.001 vs. group F, respectively

**Fig. 3:**
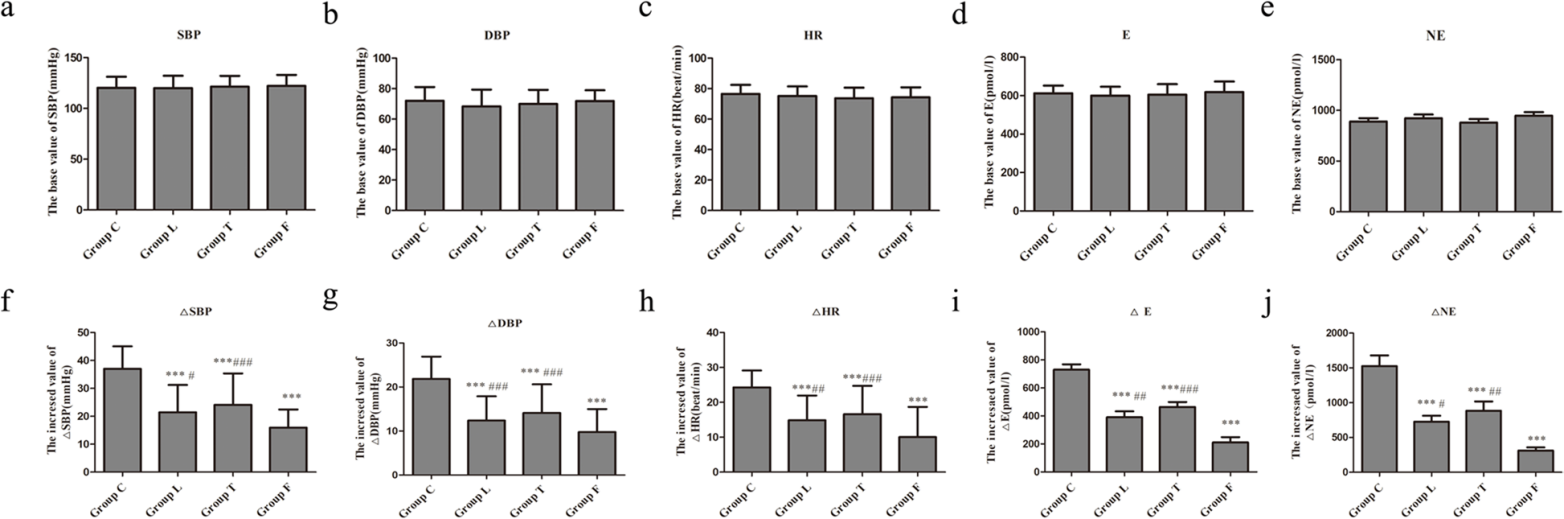
Comparison of cardiovascular measurements and serum concentrations of epinephrine and norepinephrine among groups Systolic blood pressure (SBP), diastolic blood pressure (DBP) and heart rate (HR) were measured 5 min before extubation and 1 min after extubation. Epinephrine (E) and norepinephrine (NE) plasma concentrations were assessed 1 min following extubation and at 5 min after arrival PACU. We use "△" (△ = the increased value minus the base value) to indicate the increase level for statistical analysis. **a** SBP, **b** DBP, **c** HR, **d** E, **e** NE, **f** △SBP, **g** △DBP, **h** △HR, **i** △E, **j** △NE. Data are expressed as mean ± sd. ^***^ *P* < 0.001 vs. group C; ^#^*P* < 0.05, ^##^*P* < 0.01, ^###^*P* < 0.001 vs. group F, respectively

We first assessed cough response, tracheal tube tolerance, and laryngeal discomfort. The incidence of induced coughing was significantly reduced in Group L, Group T and Group F compared to Group C (*p* < 0.001, *p* < 0.01 and *p* < 0.001, respectively). However, the incidence of coughing was significantly higher in Group L and Group T than in Group F (*p* < 0.001) (Table 2). The incidence of agitation was significantly reduced in Group L, Group T and Group F compared to Group C (*p* < 0.001, respectively). However, the incidence of agitation was significantly higher in Group L and Group T compared to Group F (*p* < 0.01, *p* < 0.001, respectively) (Table 2). The active extubation rates were significantly reduced in Group L, Group T and Group F compared to Group C (*p* < 0.01, *p* < 0.05, *p* < 0.001, respectively). However, the active extubation rate was only significantly higher in Group T compared to Group F (*p* < 0.05) (Table 2). The incidence of postoperative cough was significantly reduced in Group L, Group T and Group F compared to Group C (*p* < 0.01, *p* < 0.05, *p* < 0.01, respectively) (Table 2). We further assessed tracheal tube tolerance, and the scores were significantly improved in Group L, Group T and Group F compared to Group C (*p* < 0.001, *p* < 0.01, *p* < 0.001, receptively). However, the scores of tracheal tube tolerance were significantly higher in Group L and Group T than in Group F (*p* < 0.01, *p* < 0.001, receptively) (Fig. 2). We also evaluated the effects of tracheal tubes on the cardiovascular system. SBP, DBP, HR, E and NE at 5 min after arrival PACU were assessed as the basal levels for evaluation. The tracheal tube was removed after sputum aspiration to clear the airway, and the SBP, DBP, HR, E and NE were reassessed 1 min after extubation. The △SBP, △DBP, △HR, △E and △NE were calculated by the second levels (the level at 1 min after extubation) minus the basal levels, and these indices were used to evaluate the effect of different anaesthesia methods on airway surface anaesthesia. The basal values of SBP, DBP, HR, E, and NE were not significantly different among the groups (Fig. 3 a, b, c, d, e). However △SBP, △DBP, △HR, △E and △NE were significantly reduced in Group L, Group T and Group F compared to Group C (all *p* < 0.001) (Fig. 3 f, g, h, i, j). However, △SBP was significantly higher in Group L and Group T compared to Group F (p < 0.05, *p* < 0.001, respectively) (Fig. 3 f). △DBP was significantly higher in Group L and Group T than in Group F (*p* < 0.001, respectively) (Fig. 3 g). △HR was significantly higher in Group L and Group T than in Group F (*p* < 0.01, *p* < 0.001, respectively) (Fig. 3 h), and △E and △NE were significantly higher in Group L and Group T than in Group F (*p* < 0.05, *p* < 0.01, or *p* < 0.001) ( Fig. 3 i, j). The incidence of postoperative pharyngeal pain was significantly reduced in Group L, Group T and Group F compared to Group C (*p* < 0.05, *p* < 0.05, *p* < 0.001, respectively) (Table 2), and only the incidence of postoperative pharyngeal pain in Group F was significantly lower than Group T (*p* < 0.05) (Table 2).

**Table 2:** Comparison of cough events, tracheal tube tolerance during recovery period and postoperative cough and postoperative pharyngeal pain after operation between groups

	**Group C**	**Group L**	**Group T**	**Group F**	***χ***^***2***^***/k***	***p***
Incidence of induced coughing	51 (96.2%)	32 (61.5%) ^***###^	39 (75.0%) ^**###^	12 (22.2%%) ^***^	71.581	< 0.001
Incidence of emergence agitation	51 (96.2%)	25 (48.1%) ^***##^	32 (61.5%) ^***###^	9 (16.7%) ^***^	70.478	< 0.001
Active extubation rate	20 (37.0%)	8 (15.4%) ^**^	14 (26.9%) ^*#^	3 (5.6%) ^***^	18.575	< 0.001
Incidence of postoperative cough	21 (38.9%)	7 (13.5%) ^**^	11 (21.2%) ^*^	7 (13.0%) ^**^	14.482	0.002
Incidence of postoperative pharyngeal pain	26 (48.1%)	13 (25.0%) ^*^	18 (34.6%) ^*#^	9 (16.7%) ^***^	14.751	0.002

^*^P < 0.05, **P < 0.01, ***P < 0.001 vs. Group C; ^#^*P* < 0.05, ^##^*P* < 0.01, ^###^*P* < 0.001 vs. Group F

## Discussion

The current study compared the application of compound lidocaine/prilocaine cream, tetracaine spray or compound lidocaine/prilocaine cream combined with tetracaine to the front end of the surface of the tracheal tube and demonstrated a significant reduction in the incidence of induced coughing, agitation caused by sputum aspiration and extubation and the active extubation rate, which suggest significantly improved tolerance to the tracheal tube in patients during emergence from general anaesthesia. At least 65.1% and 75.0% of patients had induced coughing in the lidocaine/prilocaine cream and tetracaine group, respectively. However, the incidence of induced coughing was significantly reduced to 22.2% by the combination of compound lidocaine/prilocaine cream with tetracaine. The incidence of emergence agitation and active extubation rate were also significantly reduced to 16.7% and 5.6% in the compound lidocaine/prilocaine cream combined with tetracaine group, respectively. Compound lidocaine/prilocaine cream combined with tetracaine was also the most effective method to prevent extubation-induced increases in SBP, DBP, HR, E and NE plasma concentrations in patients emerging from general anaesthesia. These findings demonstrated that compound lidocaine/prilocaine cream combined with tetracaine had a better airway surface anaesthesia effect and significantly increased tolerance to the tracheal tube in patients under general anaesthesia.

Endotracheal intubation-related mechanical stimulation is clinically common and associated with airway complications including, severe coughing, laryngeal injury and postoperative sore throat [11, 12]. Coughing produces large amounts of aerosols that may contain viruses and pathogenic microorganisms, including, mycobacterium tuberculosis [13, 14], pseudomonas aeruginosa [15] and SARS-CoV-2 [16]. These aerosols containing viruses and pathogenic microorganisms may spread respiratory diseases, especially COVID-19 [4, 16]. Therefore, it is very important to prevent coughing caused by the endotracheal tube. The endotracheal tube-induced airway response (especially during intubation and extubation) also increases the risk of adverse cardiovascular events, including arrhythmias, hypertension, myocardial ischaemia, and intracranial pressure elevation [17–19].

During emergence from general anaesthesia, sputum aspiration and tracheal tube extubation are the strongest stimulators of the tracheal mucosa, which is the most likely to induce cough. Our study showed that compound lidocaine/prilocaine cream combined with tetracaine significantly reduced the incidence of induced coughing caused by sputum suction and extubation by approximately 39% compared to the use of lidocaine/prilocaine cream alone. Although the spraying of tetracaine alone also significantly reduced the incidence of cough, the reaction was lowest in the lidocaine/prilocaine cream combined with tetracaine group. One systematic review and meta-analysis of dexmedetomidine, remifentanil, fentanyl, lidocaine i.v., intracuff lidocaine, and lidocaine via tracheal or topical route, demonstrated that in pair-wise comparisons all study medications were equivalent in reducing moderate and severe emergence coughing incidence, and were better than placebo or nothing [20]. This meta-analysis supported that lidocaine/prilocaine cream combined with tetracaine was as effective as infusions of remifentanil in reducing emergence coughing caused by extubaion. Therefore, our study provides another way for clinicians to reduce the choking reaction caused by tracheal extubation.

During emergence from general anaesthesia, the patient may exhibit emergence agitation. The main factor of emergence agitation is delayed extubation. Study showed that patients with delayed extubation developed a cough reaction was 16.7 times higher than patients without delayed extubation [21]. We found that compound lidocaine/prilocaine cream or tetracaine significantly reduced the incidence of emergence agitation by approximately 48% and 35% compared to NS, respectively. And compound lidocaine/prilocaine cream combined with tetracaine further significantly reduced the incidence of emergence agitation by approximately 16.7% and approximately 31% and 45% compared to compound lidocaine/prilocaine cream or tetracaine, respectively. The most serious effect is the voluntary removal of the endotracheal tube during recovery of general anaesthesia patients with endotracheal intubation. Our study found that compound lidocaine/prilocaine cream and tetracaine significantly reduced the active extubation rate to 15% and 26%, respectively. And compound lidocaine/prilocaine cream combined with tetracaine significantly reduced the incidence of active extubation rate to 5.6% compared to NS (37.0%). Although compound lidocaine/prilocaine cream (15.4%) compared to compound lidocaine/prilocaine cream combined with tetracaine (5.6%) was not significant different in the active extubation rate, compound lidocaine/prilocaine cream was twice as high as the compound lidocaine/prilocaine cream combined with tetracaine.

Therefore, we though that compound lidocaine/prilocaine cream combined with tetracaine inhibited the airway reflexes caused by the tracheal tube and further enhanced the body's tolerance to the tube. Our results showed that compound lidocaine/prilocaine cream or tetracaine significantly improved tracheal tube tolerance. The tracheal tube tolerance scores were reduced to 2 (0, 1) and 3 (0, 1.25), respectively. Compound lidocaine/prilocaine cream combined with tetracaine further significantly increased tracheal tube tolerance, and the tracheal tube tolerance score was reduced to 1 (0, 0). Study showed that remifentanil 0.025–0.05 microg.kg(-1).min(-1) achieves satisfactory tracheal tube tolerance in awake and spontaneously breathing patients performed under general anaesthesia, the respiratory response subscore of comfort scale of patients is all 3 [22]. Our findings also provide a new procedure to increase tracheal tube tolerance in patients with general anaesthesia in PACU.

These findings may induce beneficial effects on cardiovascular reactions during the course of extubation. The current study found that hemodynamic changes (blood pressure and/or HR) exceeded 20% of the baseline at 1 min after extubation. Compound lidocaine/prilocaine cream or tetracaine significantly reduced the SBP increase from baseline (△SBP), the DBP increase from baseline (△DBP) and the HR increase from baseline (△HR) about 8% ~ 12% compared to the use saline. However, compound lidocaine/prilocaine cream combined with tetracaine significantly reduced the average values of △SBP, △DBP and △HR about 30% ~ 50% compared to the use saline.

When the endotracheal tube is removed, this stimulates the receptors of the airway mucosa, which results in the release of catecholamines [23, 24] and consequent increases in blood pressure and heart rate. Attari et al. used labatolol [25] and Zhao et al. used remifentanil [18] to inhibit the increase in blood pressure caused by extubation and reported similar results as our study. We found that catecholamine (E and NE) changes 1 min after extubation were 1.1 to 1.7 times baseline levels in the control group. Compound lidocaine/prilocaine cream or tetracaine significantly reduced the increases in E and NE (△E and △NE) by approximately 50%. Compound lidocaine/prilocaine cream combined with tetracaine further significantly reduced the average values of △E and △NE by an additional 50%. Jiang M, etal suggested that intravenous oxycodone reduced the increased average levels of △E and △NE by approximately 87% and 93%, respectively, compared to intravenous saline injection [10], and these results are similar to our study. These results may be useful in preventing cardiovascular events during emergence from general anaesthesia.

We also assessed the incidence of postoperative cough and postoperative pharyngeal pain. We found that the use of local anaesthetic significantly reduced these incidences compared to saline. The incidence of postoperative cough in the compound lidocaine/prilocaine cream (13.5%), tetracaine (21.2%) and compound lidocaine/prilocaine cream combined with tetracaine (13.0%) groups were not significantly different from each other. However, the incidence of postoperative pharyngeal pain was higher in the tetracaine group than in the compound lidocaine/prilocaine cream combined with tetracaine group. Soltani, H.A. and O. Aghadavoudi suggested that the use of lidocaine to inflate the endotracheal tube cuff at the end of surgery decreased the frequency of postoperative cough and sore throat by approximately 41.4% and 17.7% respectively, compared to the use of saline to inflate the endotracheal tube cuff [26]. Their results were similar to our study in reducing the magnitude of the incidence.

There are some potential risks in the application of local anaesthetics to the airway of a patient who is under general anaesthesia: 1) if the operation time is too long, the effect of the local anaesthetic will insufficient to suppress the cough response caused by extubation; 2) the local anaesthetic effects may extend to block part of the vocal cords and affect vocalisation; 3) compound lidocaine includes prilocaine, although we used safe doses, prilocaine may increase blood methemoglobin levels, which should be monitored in clinical practice.

And there are some limitations to our study. 1) This study did not observe the duration of the continuous effect of compound lidocaine combined with tetracaine on airway topical anaesthesia. 2) This study did not consider the patient's anxiety state, which will affect the release of catecholamines during awakening, and may affect the evaluation of catecholamines released during tracheal tube extubation. 3) During recovery from general anaesthesia, the patient exhibits emergence agitation due to poor tolerance to the tracheal tube, which could not be well revealed in this study based on other factors, such as preoperative anxiety or bladder irritation caused by urine retention.

## Conclusion

In summary, we demonstrated that the application of compound lidocaine/prilocaine cream combined with tetracaine to the surface of the tracheal tube prevented the cough caused by extubation, increased the body's tolerance to the tracheal tube and reduced the release of catecholamine hormones into the blood and the increase in blood pressure during extubation in patients undergoing laparoscopic cholecystectomy and cholecystectomy combined with biliary exploration. Therefore, compound lidocaine cream combined with tetracaine may be a more effective approach for preventing coughing and stabilising circulation during extubation following emergence from general anaesthesia. This may play an important role in preventing medical staff from contracting respiratory infectious diseases, especially during the COVID-19 epidemic.

## Data Availability

The datasets generated and/or analysed during the current study are not publicly available due to limitations of ethical approval involving the patient data and anonymity but are available from the corresponding author on reasonable request. The corresponding author: Prof. *Erfei Zhang*, Department of Anesthesiology, The Affiliated Hospital of Yan’an University, Yan’an 716,000, Shaanxi Province, P. R. China). Tel: + 86 0911 2,881,264, e-mail: zhangerfei09@126.com) on reasonable request.
